# Geraniin attenuates LPS-induced acute lung injury via inhibiting NF-κB and activating Nrf2 signaling pathways

**DOI:** 10.18632/oncotarget.15227

**Published:** 2017-02-09

**Authors:** Guangfa Zhu, Xi Xin, Yan Liu, Yan Huang, Keng Li, Chunting Wu

**Affiliations:** ^1^ Department of Pulmonary and Critical Care Medicine, Beijing Anzhen Hospital, Capital Medical University, Beijing Institute of Heart, Lung and Blood Vessel Diseases, Beijing 100029, P.R. China; ^2^ Department of Infectious Diseases, Beijing Anzhen Hospital, Capital Medical University, Beijing Institute of Heart, Lung and Blood Vessel Diseases, Beijing 100029, P.R. China

**Keywords:** geraniin, LPS, Nrf2, lung injury

## Abstract

Geraniin, a typical ellagitannin isolated from *Phyllanthusurinaria Linn*, has been reported to have anti-inflammatory effect. The aim of the study is to investigate the therapeutic effects of geraniin on LPS-induced acute lung injury (ALI) in mice. The mice were intranasal adminisration of LPS for 12 h. Geraniin was intra-peritoneal injection 1 h after LPS treatment. The results showed that geraniin significantly attenuated LPS-induced pathological changes in the lung. Geraniin also inhibited LPS-induced macrophages and neutrophils infiltration in the lung. Geraniin significantly attenuated LPS-induced elevation of MPO level. LPS-induced TNF-α, IL-6 and IL-1β production were markedly suppressed by treatment of geraniin. Furthermore, geraniin inhibited NF-κB activation in LPS-induced ALI. In addition, geraniin was found to up-regulate the expression of Nrf2 and HO-1. In conclusion, these data suggested that geraniin had therapeutic effects in LPS-induced ALI by inhibiting NF-κB and activating Nrf2 signaling pathways.

## INTRODUCTION

Acute lung injury (ALI) is a critical pulmonary inflammatory disease that characterized by progressive hypoxemia, edema, and neutrophil accumulation in the lung [1]. Previous studies have suggested that Lipopolysaccharide (LPS) was the major stimulus of ALI and inflammation played a critical role in the pathogenesis of ALI [2]. LPS could induce the production of inflammatory mediators via NF-κB signaling pathway [3]. These inflammatory mediators amply the inflammatory response and lead to lung injury [4]. ALI is often associated with high morbidity and mortality in ill patients [5, 6]. Despite recent advances in intense care research of acute lung injury, its incidence remains high [7, 8]. Therefore, an effective therapeutic option against ALI drug is urgently required. Nrf2, an important transcription factor, can be activated by redox-dependent stimuli. Previous studies showed that activating Nrf2 could regulate inflammatory and oxidative stress [9]. Furthermore, activating Nrf2 could inhibit lung injury [10]. Therefore, Nrf2 may used as a target in the treatment of lung injury.

Geraniin, a typical ellagitannin extracted from *Phyllanthusurinaria Linn*, has been reported to have anti-oxidant and anti-inflammatory activities [11]. Geraniin was found to inhibit LPS-induced inflammation in RAW264.7 cells [12]. Also, geraniin was found to inhibit LPS-induced THP-1 macrophages switching to M1 phenotype [13]. Furthermore, it has been reported that geraniin protected cells from H_2_O_2_-induced oxidative cell death [14]. In addition, studies showed that geraniin protected liver cells against ethanol induced cytotoxicity [15]. However, whether geraniin could protect against LPS-induced acute lung injury was uncertain. Therefore, we investigated the protective effects of geraniin on LPS-induced ALI in mice.

## RESULTS

### Geraniin reduces inflammatory cells infiltration in LPS-induced lung injury

**Figure 1:**
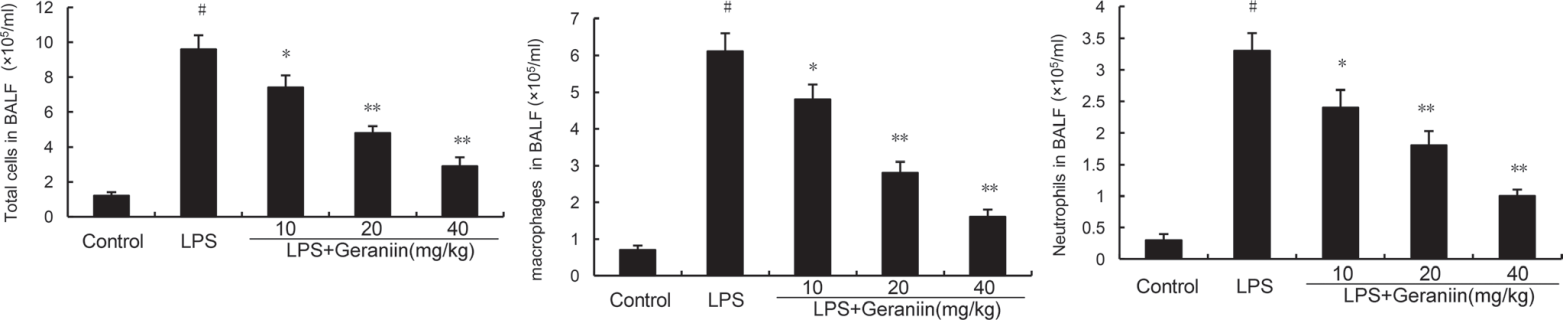
Effects of geraniin on inflammatory inflammatory cells infiltration in the BALF 12 h after LPS treatment, the BALF were collected and the numbers of inflammatory cells were detected. The values presented are the mean ± SEM of three independent experiments. ^#^*p* < 0.01 vs. control group, *p < 0.05, ^*^p < 0.01 vs. LPS group.

The effects of geraniin on inflammatory cells infiltration were measured in this study. As shown in Figure 1, compared with the control group, LPS significantly increased the numbers of total cells, neutrophils and macrophages. However, this increases induced by LPS was reduced with the administration of geraniin in a dose dependent manner.

### Effects of Geraniin on LPS-induced lung histopathological changes

**Figure 2:**
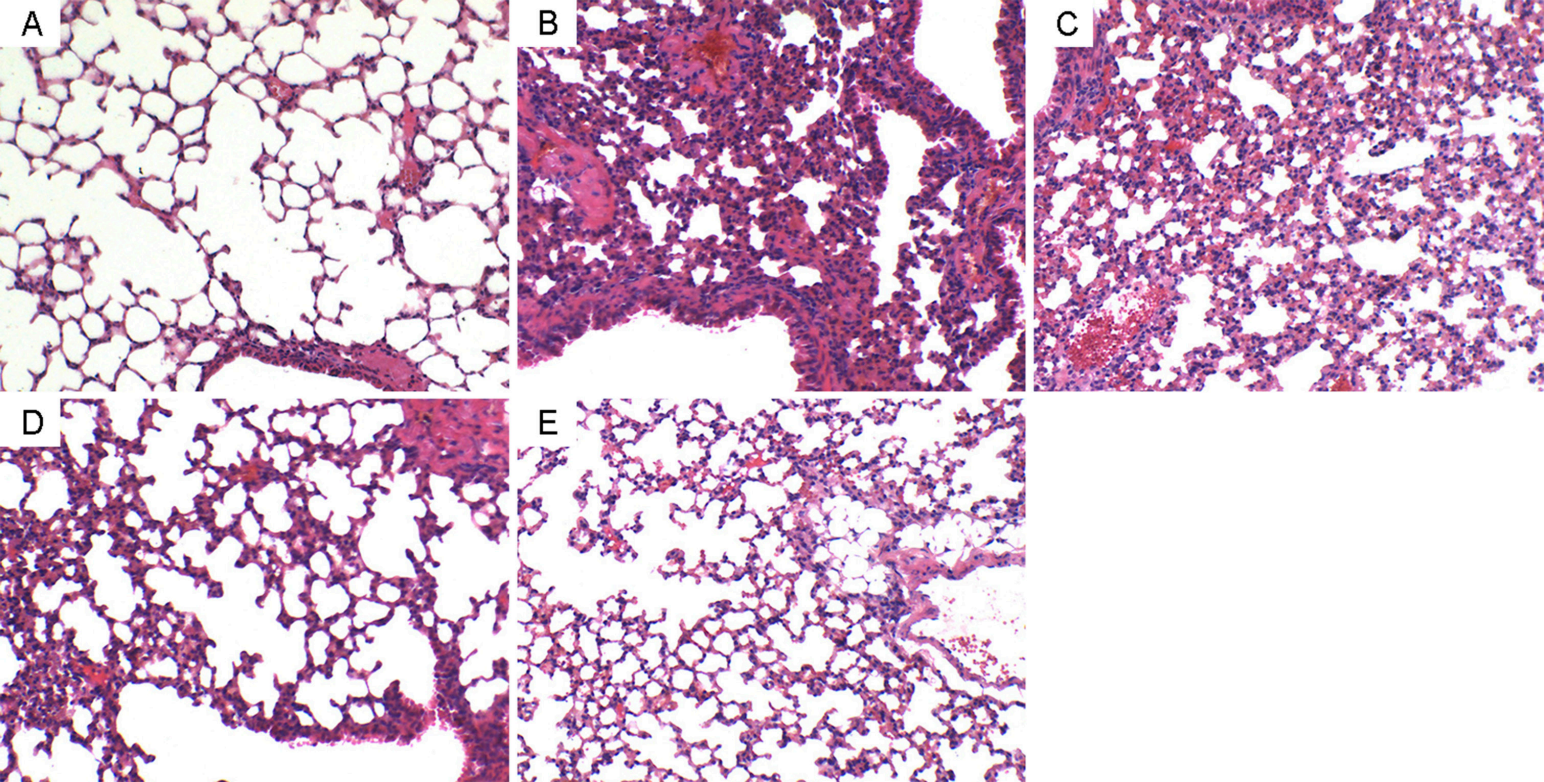
Effects of geraniinon histopathological changes in lung tissues in LPS-induced ALI mice Representative histological changes of lung obtained from mice of different groups. (**A**) Control group, (**B**) LPS group, (**C**) LPS+ geraniin (10 mg/kg) group, (**D**) LPS+geraniin (20 mg/kg) group (**E**) LPS+geraniin (40 mg/kg) group (Hematoxylin and eosin staining, magnification 200×).

In this study, we detected the effects of geraniin on LPS-induced lung histopathological changes (Figure 2A). In the LPS group without treatment of geraniin, lung tissues showed evident histopathological abnormalities, evidenced by the presence of alveolar wall thickness, airalveoli, emphysematous and pulmonary congestion (Figure 2B). However, compared with LPS group, the histopathological changes of lung tissues were improved by geraniin administration (Figure 2C–2E).

### Effects of geraniin on MOP activity and lung W/D ratio

**Figure 3:**
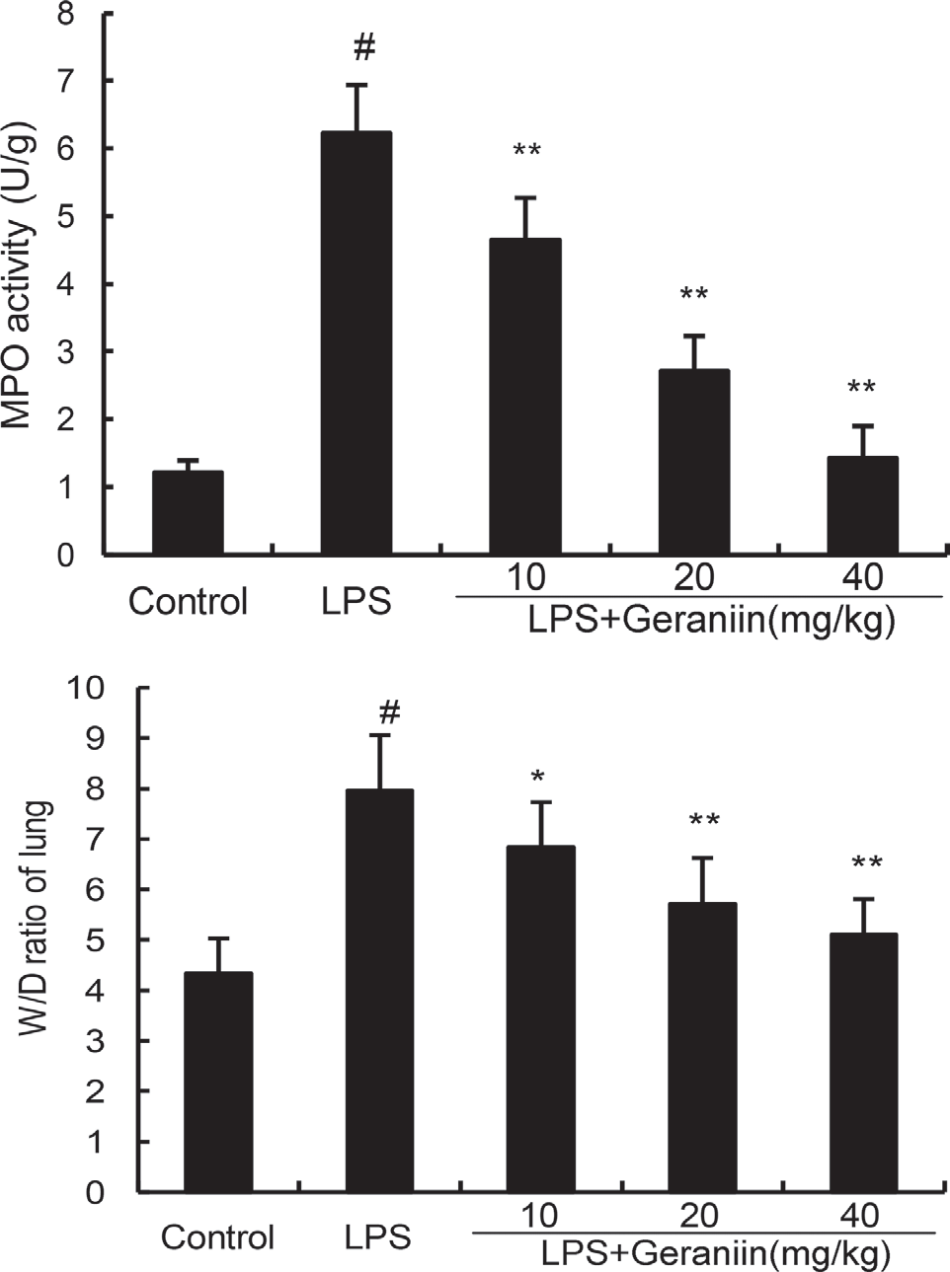
Effects of geraniin on MPO activity and lung W/D ratioof LPS-induced ALI 12 h after LPS treatment, the lung tissues were collected and MPO activity was detected. The values presented are the mean ± SEMof three independent experiments. ^#^*p* < 0.01 vs. control group, *p < 0.05, ^*^p < 0.01 vs. LPS group.

MPO activity, a biomarker of neutrophils, was detected in this study. As shown in Figure 3, compared with the control group, LPS significantly increased MPO activity in lung tissues. However, this increase induced by LPS was reduced with the administration of geraniin in a dose dependent manner. The effects of geraniin on LPS-induced lung W/D ratio were also measured. The results showed that LPS significantly increased lung W/D ratio. However, geraniin significantly inhibited LPS-induced lung W/D ratio (Figure 3).

### Effects of geraniin on cytokine production in BALF

**Figure 4:**
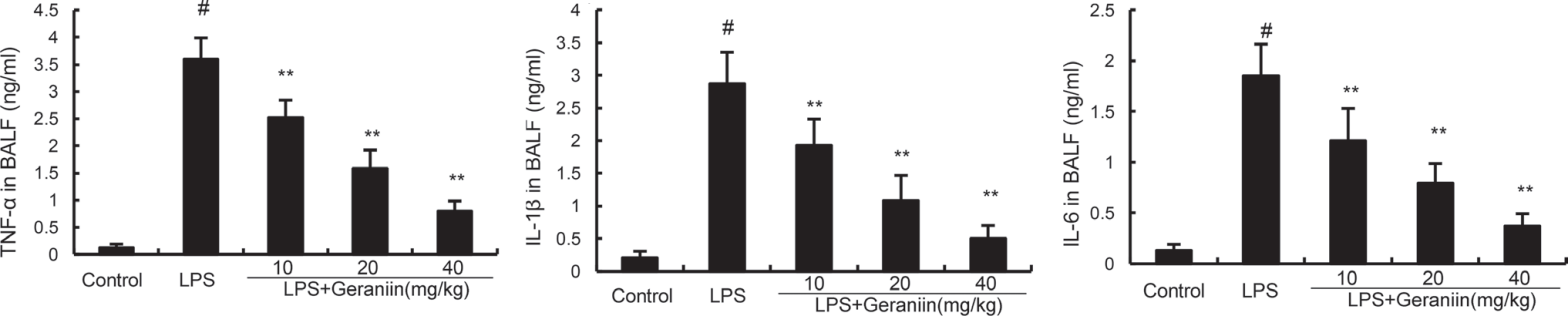
Effects of geraniin on TNF-α, IL-1ß, and IL-6 production in the BALF of LPS-induced ALI mice 12 h after LPS treatment, the BALF were collected and the levels of TNF-α, IL-1ß, and IL-6 were detected. The values presented are mean ± SEM of three independent experiments. ^#^*p* < 0.01 vs. control group, *p < 0.05, ^*^p < 0.01 vs. LPS group.

BALF was collected to determine the effects of geraniin on LPS-induced inflammatory cytokines production. Compared with the control group, the results showed that LPS significantly increased the levels of TNF-α, IL-1β, and IL-6 in BALF. However, treatment of geraniin dose-dependently reduced TNF-α, IL-1β, and IL-6 production compared with LPS group (Figure 4).

### Effects of geraniin on LPS-induced NF-κB activation

**Figure 5:**
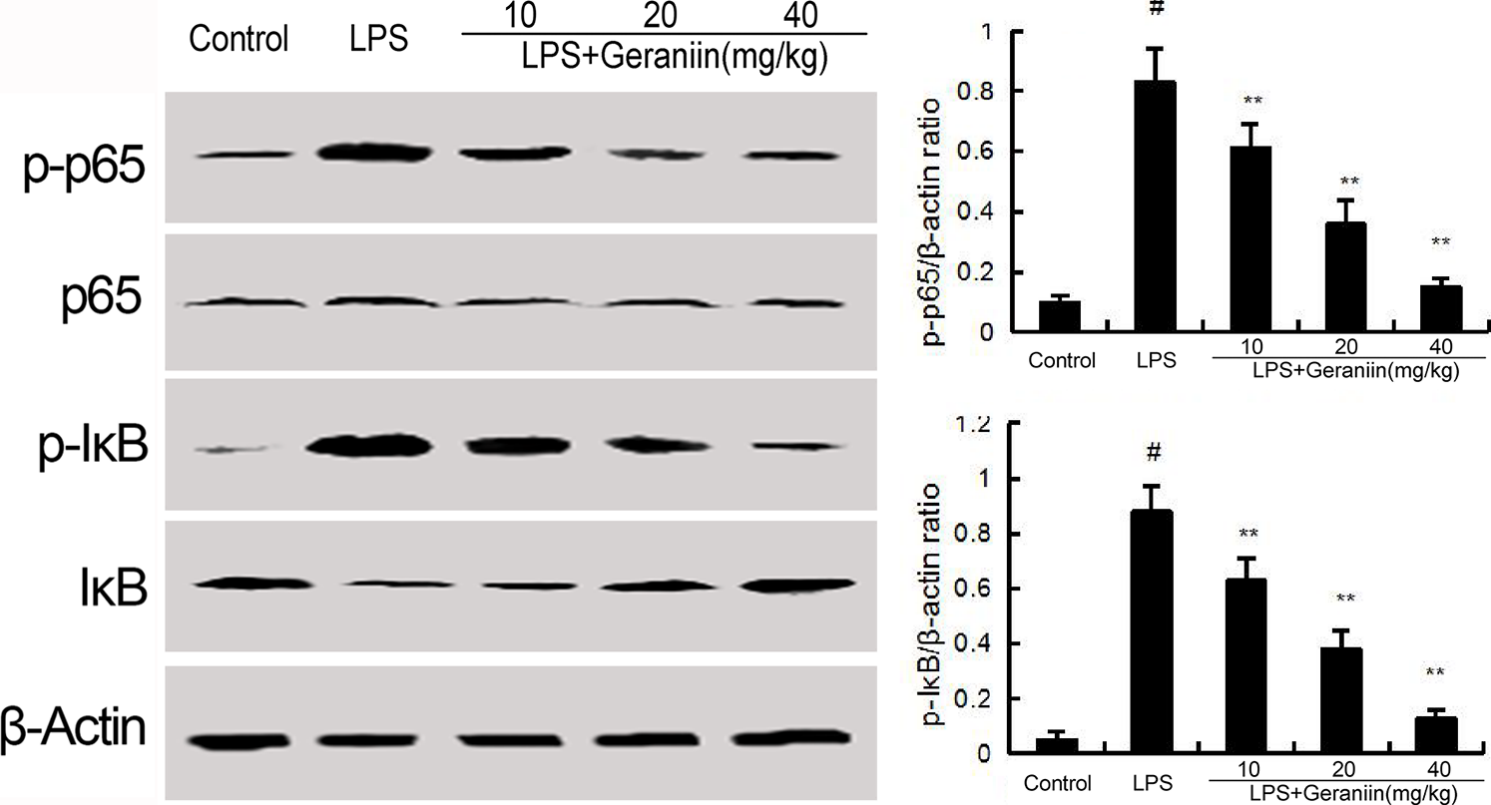
Geraniin inhibits LPS-induced NF-κB activation The values presented are the means ± SEM of three independent experiments. ^#^*p* < 0.01 vs. control group, *p < 0.05, ^*^p < 0.01 vs. LPS group.

Western blot were performed to determine the phosphorylation of NF-κB p65 and IκBα. In this study, we found that LPS increased the phosphorylation of NF-κB p65 and IκBα. However, treatment with geraniin decreased the phosphorylation of NF-κB and IκBα (Figure 5).

### Effects of geraniin on HO-1 and Nrf2 expression

**Figure 6:**
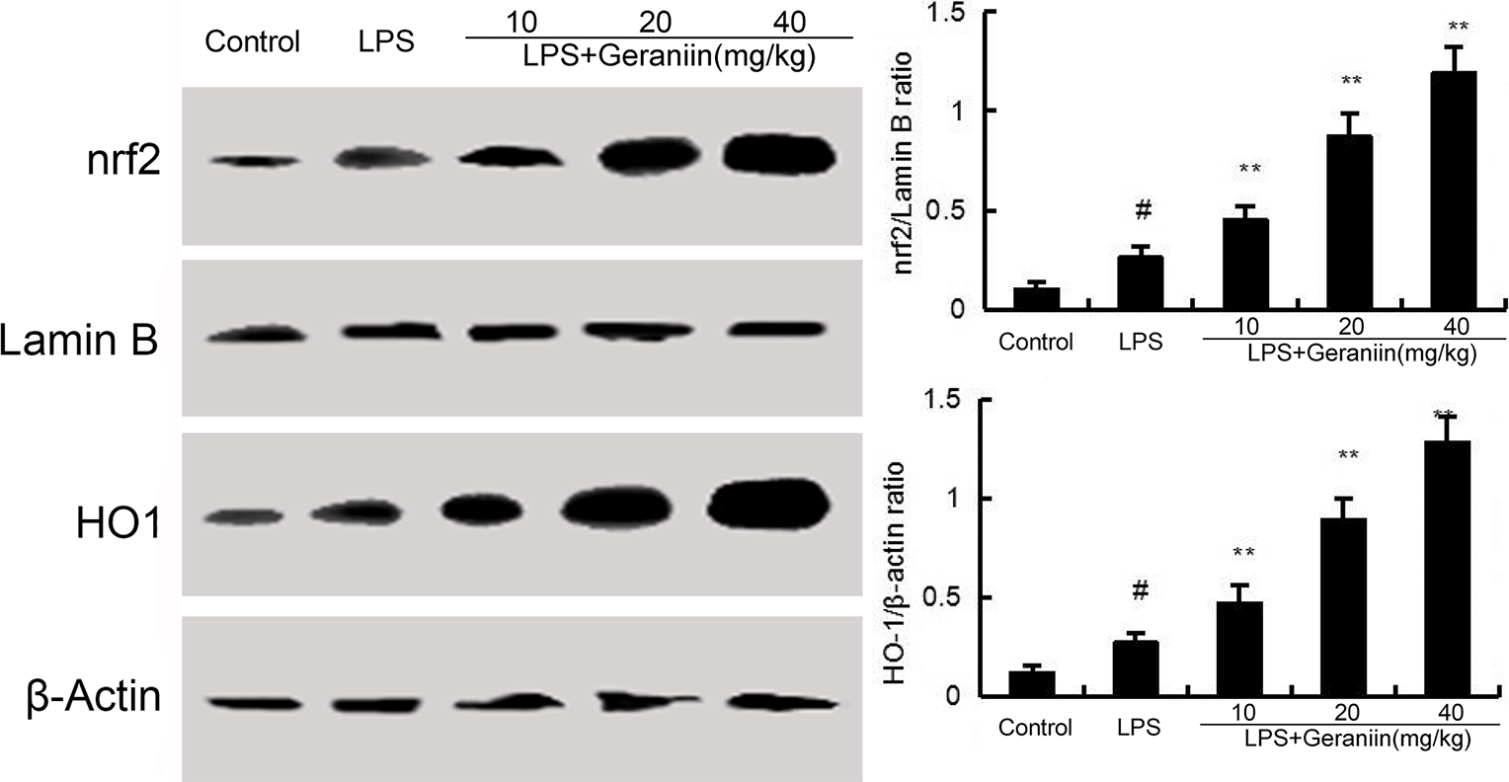
Effects of geraniin on Nrf2 signaling pathway The values presented are the means ± SEM of three independent experiments. ^#^*p* < 0.01 vs. control group, *p < 0.05, ^*^p < 0.01 vs. LPS group.

To investigate the anti-inflammatory mechanism of geraniin, the effects of geraniin on Nrf2 signaling pathway were detected. Compared with the control group, increased the expression of Nrf2 and HO-1. However, treatment of geraniin dose-dependently up-regulated the expression of Nrf2 and HO-1 (Figure 6).

## DISCUSSION

In this study, we report for the first time that geraniinhas therapeutic effect against LPS-induced ALI. Geraniin attenuates LPS-induced ALI by inhibiting inflammatory mediators production. The pharmacological actions of geraniin were associated with the activation of Nrf2 and inhibition of NF-κB signaling pathways.

In LPS-induced ALI, exposure of lungs to LPS leads to the release of inflammatory cytokines, such as TNF-α, IL-1β, IL-6 via NF-κB signaling pathway [16]. These inflammatory cytokines amply the inflammatory response and lead to the pathogenesis of ALI [17]. Furthermore, these inflammatory cytokines could induce the infiltration of neutrophilsand damage alveolar epithelial permeability and lead to lung edema [18, 19]. Previous studies showed that inhibition of these inflammatory cytokines could attenuate the pathogenesis of ALI [20]. In this study, we found that geraniin significantly inhibited LPS-induced inflammatory cytokines production. In addition, the infiltration of neutrophils and lung edema were also suppressed by treatment of geraniin. These results indicated that geraniin protected against LPS-induced ALI by inhibiting inflammatory cytokines production.

A growing body of studies showed that NF-κB signaling pathway played an important role in the pathophysiology of ALI [21]. In our previous study, we found that inhibition of NF-κB could protect sepsis-induced ALI in mice [22]. Normally, NF-κB is located in the cytoplasm with inhibitory protein IκB. LPS could induce NF-κB p65 dissociates from IκB. Then, NF-κB p65 translocates into the nucleus to induce the expression of inflammatory cytokines [23, 24]. Inhibiting of NF-κB signaling pathway could attenuate LPS-induced ALI by inhibiting inflammatory cytokines production [25, 26]. To clarify the anti-inflammatory mechanism of geraniin, the effects of geraniinon NF-κB activation were measured. The results suggested that geraniin significantly inhibited LPS-induced NF-κB activation. The transcription factor Nrf2 regulates the expression of antioxidant genes [27] and previous studies suggested that Nrf2 affected the severity of acute lung injury [28]. Studies showed that deletion of Nrf2 in airway epithelium exacerbated acute lung injury and attenuated the inflammatory response [29]. In addition, it has been reported that activation of Nrf2 could attenuate LPS-induced lung injury [30]. In previous study, our results showed that eriodictyol protected LPS-induced ALI by activating Nrf2 [31]. These results suggested that Nrf2 had protective effects against acute lung injury. In this study, our results showed that geraniin up-regulated the expression of Nrf2 and HO-1 in a dose dependent manner. These results suggested that geraniin protected against LPS-induced lung injury by activating Nrf2 signaling pathway.

In conclusion, our results showed that geraniin had therapeutic effects against LPS-induced ALI. The beneficial effects of geraniin are due to its anti-inflammatory effects. Geraniin exhibited anti-inflammatory effects by inhibiting NF-κB and activating Nrf2 signaling pathways. Geraniin may serve as a novel agent for the treatment of ALI.

## MATERIALS AND METHODS

### Reagents

Geraniin (purity > 98%) was purchased from the Chinese Institute for Drug and Biological Product Control (Beijing, China). LPS was purchased from Sigma Chemical Co. (St. Louis, MO, USA). Mouse TNF-α,IL-6 and IL-1βELISA kits were obtained from BioLegend (CA, USA). Antibodies for Nrf2, HO-1, NF-κB p65, IκBα, and β-actin were purchased from CST (Danvers, MA, USA).

### Animals

BALB/c mice (6–8 weeks) were purchased from the Center of Experimental Animals of Capital Medical University (Beijing, China). The mice were received a standard diet and housed in a room with controlled temperature. The mice were adapted the environment for 7 days. All animal experimental procedures were approved by the Committee of Animal Experimentation of the Capital Medical University.

### Experimental design

Sixty mice were randomly divided into five groups and each group contains 12 mice. Group A, the control group; group B, LPS group; group C to E, LPS+geraniin (10, 20 and 40 mg/kg) groups. The mice of Group B were received with 50 μL LPS given byintranasally (i.n.). The mice of C to E groups were received with 50μL LPS given byintranasally (i.n.). 1 h later, geraniin (10, 20 and 40 mg/kg) was given to mice. The doses of geraniin were based on previous study [32]. The mice of Group A were given equal amount of PBS. 12 h after LPS treatment, the mice were killed and the BALF were collected. We chose 12 h after LPS treatment was based on previous studies [33].

### Histopathologic evaluation of lung tissues

Lung tissue was fixed with 10% buffered formalin for 12 h, paraffin embedded and cut into 5-μm sections. After the section were stained with hematoxylin and eosin (H&E), the sections were observed with a microscope (Olympus, Tokyo, Japan), as described previously.

### BALF and cell count

12 h after LPS treatment, the mice were killed and the BALF were collected. The number of total cells was counted using a hemocytometer. The numbers of neutrophils and macrophages in BALF were stained with the Kwik-Diff staining set (Thermo, USA).

### MPO assay

MPO activity was measured to demonstrate the neutrophil infiltration using the myeloperoxidase fluorometric activity assay kit (Sigma). 12 h after LPS challenge, the mice were killed and the lung tissues were collected and homogenized in cool normal saline. Then the MPO activity was measured using test kits purchased from multi-mode microplate reader (BioTek, SynergyHT, Bedfordshire, United Kingdom).

### Inflammatory cytokines assay

Specific ELISA kits (BioLegend, CA, USA) were used to quantify TNF-α, IL-1β, and IL-6 in the BALF. The standard curve was established according to the standards provided by the kits.

### Lung W/D ratio

The inferior lobe of the right lung was collected to obtain the ‘wet’ weight. Then, the lung was dried at 80°C for 48 h to obtain the ‘dry’ weight. The lung W/D ratio was calculated by dividing the lung wet weight by the lung dry weight.

### Western blot analysis

Total proteins from lung microglia were extracted by M-PER Mammalian Protein Extraction reagent (Thermo, USA). Samples with equal amounts (40 μg) of protein were fractionated on 10% SDS-polyacrylamide gel and transferred on nitrocellulose membranes. The membranes were blocked with 5% bovine serum albumin. Then, the membranes were probed with the primary antibodies and secondary antibodies at room temperature. The proteins were measured using the ECL detection reagents (BioLegend, CA, USA).

### Statistical analyses

Data are expressed in terms of mean ± SD. Data were analyzed by using one-way analysis of variance followed by post-hoc Dunnett's test with a significancelevel of *p* < 0.05. Statistical analyseswere performed using the SPSS software package.
